# Asthma and asthma symptom control in relation to incidence of lung cancer in the HUNT study

**DOI:** 10.1038/s41598-021-84012-3

**Published:** 2021-02-25

**Authors:** Lin Jiang, Yi-Qian Sun, Arnulf Langhammer, Ben Michael Brumpton, Yue Chen, Tom IL Nilsen, Linda Leivseth, Sissel Gyrid Freim Wahl, Xiao-Mei Mai

**Affiliations:** 1 Department of Public Health and Nursing, Faculty of Medicine and Health Science, Norwegian University of Science and Technology (NTNU), Postbox 8905, MTFS, N-7491, Trondheim, Norway; 2 Department of Clinical and Molecular Medicine, Faculty of Medicine and Health Science, Norwegian University of Science and Technology, Trondheim, Norway; 3 Department of Pathology, Clinic of Laboratory Medicine, St. Olavs Hospital, Trondheim University Hospital, Trondheim, Norway; 4 TkMidt-Center for Oral Health Services and Research, Mid-Norway, Trondheim, Norway; 5 HUNT Research Centre, Department of Public Health and Nursing, Norwegian University of Science and Technology, Levanger, Norway; 6 Levanger Hospital, Nord-Trøndelag Hospital Trust, Levanger, Norway; 7 Clinic of Thoracic and Occupational Medicine, St. Olavs Hospital, Trondheim University Hospital, Trondheim, Norway; 8 K.G. Jebsen Centre for Genetic Epidemiology, Department of Public Health and Nursing, Norwegian University of Science and Technology, Trondheim, Norway; 9 MRC Integrative Epidemiology Unit, University of Bristol, Bristol, UK; 10 School of Epidemiology and Public Health, Faculty of Medicine, University of Ottawa, Ottawa, Canada; 11 Clinic of Anesthesia and Intensive Care, St. Olavs Hospital, Trondheim University Hospital, Trondheim, Norway; 12 Centre for Clinical Documentation and Evaluation (SKDE), Northern Norway Regional Health Authority, Tromsø, Norway

**Keywords:** Diseases, Oncology, Risk factors

## Abstract

Large prospective studies on asthma, especially asthma symptom control, as a potential risk factor for lung cancer are limited. We followed up 62,791 cancer-free Norwegian adults from 1995–1997 to 2017. Self-reported doctor-diagnosed asthma was categorized into active and non-active asthma. Levels of asthma symptom control were classified into controlled and partially controlled (including partly controlled and uncontrolled) according to the Global Initiative for Asthma guidelines. Incident lung cancer cases were ascertained from the Cancer Registry of Norway. Cox regression models were used to estimate hazard ratios (HRs) with 95% confidence intervals (CIs) for possible associations. Totally, 984 participants developed lung cancer during a median follow-up of 21.1 years. After adjustment for smoking and other potential confounders, an increased incidence of lung cancer was found for adults with partially controlled asthma (HR 1.39, 95% CI 1.00–1.92) compared with those without asthma at baseline. Adults with active asthma had a tendency of increased lung cancer incidence (HR 1.29, 95% CI 0.95–1.75). Sensitivity analyses indicated that the observed associations were less likely resulted from reverse causation or residual confounding by smoking. Our findings suggested that proper control of asthma symptoms might contribute to a reduced incidence of lung cancer.

## Introduction

Asthma is a common chronic lung disease characterized by chronic inflammation, reversible airway obstruction and enhanced bronchial reactivity^1,2^. The chronic inflammatory state of the lung among those with asthma has been suggested to cause oxidative injuries that may lead to lung cancer development^3^.

Given the relatively high prevalence of asthma and low survival rate of lung cancer worldwide, it is important to clarify if asthma is a risk factor for lung cancer. So far, three meta-analysis studies have investigated the possible association between asthma and lung cancer and a positive association has been suggested^2,4,5^. More case–control studies than prospective studies were included in the meta-analysis studies. Among the included prospective studies, several used age and sex-standardized incidence ratio (SIR) to present the relative risk for lung cancer in patients with asthma compared with a general population^6–8^. Smoking, the most important confounding factor, was not controlled for. Also, the included prospective cohort studies tended to have inadequate number of lung cancer cases due to short follow-up duration (< 10 years)^9,10^ or small sample size^11,12^. A recent prospective cohort study using data from the UK Million Women Study with more than 14 years of follow-up reported that asthma requiring treatment was associated with an increased incidence of lung cancer in never smoking women^13^; however, it investigated 34 potential risk factors simultaneously and a chance finding could not be excluded.

Asthma is not curable, but it is controllable with appropriate treatment. Nonetheless, about half of people with asthma in Europe are reported to be either partly controlled or uncontrolled^14,15^. Failure to control asthma symptoms may not only increase asthma exacerbations but also increase risk of other disease such as atrial fibrillation^16^. Few studies have investigated the possible associations between the levels of asthma symptom control and lung cancer risk.

The aim of the current study was to explore the potential associations of asthma overall as well as asthma status and symptom control with lung cancer incidence in a large prospective cohort study with a long follow-up duration.

## Results

In total, 984 of the 62,791 participants developed lung cancer during a median follow-up of 21.1 years. Table 1 describes the distribution of baseline characteristics of participants by asthma categories. Compared to those without asthma, participants with non-active or active asthma (active asthma was defined if participants with asthma confirmed symptoms of wheezing or reported using asthma medication at the baseline survey) were more likely to be former or passive smokers, and to have allergic rhinitis, economic difficulties or a family history of cancer at baseline (Table 1). Similar patterns were found among participants with controlled or partially controlled asthma (the latter was defined as fulfilling one or more items based on the Global Initiative for Asthma guidelines) compared with participants without asthma (Supplementary Table S1). The proportion of controlled asthma was 37% among the participants with asthma and was 29% among the participants with active asthma.

**Table 1:** Distribution of baseline characteristics according to asthma categories in the HUNT2 Study, 1995–1997 (n = 62,791).

Variables	No asthma	Non-active asthma	Active asthma
**Number of subjects**	**59,591**	**1090**	**2110**
Age (years)	49.5 ± 17.0	47.3 ± 17.2	51.1 ± 17.3
Body mass index (kg/m^2^)	26.3 ± 4.0	26.7 ± 4.4	27.4 ± 4.9
**Number of lung cancer cases (%)**	**921 (1.6)**	**14 (1.3)**	**49 (2.3)**
Sex, % (women/men)	53.0/47.0	51.2/48.8	56.2/43.8
Allergic rhinitis, % (no/yes/unknown)	70.5/5.5/24.0	40.2/45.8/14.0	35.6/50.0/14.4
Smoking status, % (never/current/former/unknown)	43.0/28.6/26.3/2.2	40.6/28.2/29.0/2.2	37.3/28.5/32.0/2.1
Passive smoking, % (never/ever/unknown)	18.5/79.6/1.9	15.9/83.0/1.1	14.7/83.2/2.0
Alcohol consumption (times/month), % (never/ ≥ 1/unknown)	34.6/56.9/8.6	33.9/58.0/8.2	39.0/52.4/8.2
Physical activity, % (inactive^1^/active^2^/unknown)	21.6/48.0/30.4	25.4/50.4/24.2	22.4/45.9/31.7
Total sitting time daily (hours), % (< 8/ ≥ 8/unknown)	48.1/27.8/24.1	52.2/35.2/12.6	54.6/31.9/13.6
Education (years), % (< 10/ ≥ 10/unknown)	34.0/61.0/5.1	31.8/63.1/5.1	38.5/55.5/6.0
Economic difficulties, % (no/yes/unknown)	48.0/21.1/30.9	51.5/28.1/20.5	47.4/31.1/21.5
Family history of cancer, % (no/yes)	75.0/25.0	72.8/27.2	69.1/30.9

HUNT: Nord-Trøndelag Health Study.

Data are given as mean ± standard deviation or percentage of subjects in each asthma category.

^1^Inactive: no physical activity or only light physical activity ≤ 2 h per week.

^2^Active: physical activity level from low to high.

There was no clear association between asthma overall and lung cancer incidence, with a HR of 1.19 (95% CI 0.91–1.57) after adjustment for smoking and other confounders (Table 2). Active asthma tended to be associated with an increased lung cancer incidence with an imprecise estimate (HR 1.29, 95% CI 0.95–1.75). Notably, partially controlled asthma showed an increased lung cancer incidence with an adjusted HR of 1.39 (95% CI 1.00–1.92). Neither non-active asthma nor controlled asthma was associated with the incidence of lung cancer (Table 2). In the adjusted model allergic rhinitis, as a possible confounder, was not associated with lung cancer incidence (HR 1.05, 95% CI 0.80–1.39). In addition, participants with both asthma and allergic rhinitis had a similar HR (1.25, 95% CI 0.86–1.82) for lung cancer incidence as participants with asthma and without allergic rhinitis (1.22, 95% CI 0.81–1.83) compared with those without asthma.

**Table 2:** The associations of asthma overall, asthma status and levels of asthma symptom control with lung cancer incidence, the HUNT Study, 1995–97 to 2017 (n = 62,791).

					Crude^1^		Adjusted^2^	
Asthma overall			n/Cases	IR (per 1000 person-years)	HR	95% CI	HR	95% CI
No			59,591/921	0.82	1.00	Reference	1.00	Reference
Yes			3200/63	1.08	1.29	1.00–1.67	1.19	0.91–1.57
	Asthma status							
		Non-active asthma	1090/14	0.69	1.01	0.59–1.71	0.94	0.55–1.61
		Active asthma	2110/49	1.28	1.41	1.06–1.88	1.29	0.95–1.75
	Asthma symptom control^3^							
		Controlled	1170/15	0.66	0.88	0.53–1.46	0.91	0.54–1.52
		Partially controlled	1622/42	1.47	1.58	1.16–2.16	1.39	1.00–1.92

CI: confidence interval; HR: hazard ratio; IR: incidence rate.

^1^Age was used as the time scale in the crude model.

^2^Adjusted for sex, body mass index, smoking [(never, former (< 10, 10–20, and > 20 pack-years (pyrs)), current (< 10, 10–20, and > 20 pyrs)], passive smoking, alcohol consumption, physical activity, total sitting time daily, education, economic difficulties, family history of cancer and allergic rhinitis. Age was used as the time scale. *Tvc* option of the *stcox* command in Stata was used to model the non-proportional hazards for sex, smoking and economic difficulties in the adjusted models.

^3^An “unknown” level of asthma symptom control is not shown due to limited lung cancer cases (n = 6).

Results from the following sensitivity analyses provided supportive evidence for the above findings: (1) After excluding the first three-year follow-up, the associations of active asthma and partially controlled asthma with lung cancer incidence were strengthened (HR 1.41, 95% CI 1.04–1.93 for active asthma and HR 1.54, 95% CI 1.10–2.14 for partially controlled asthma, Table 3). After exclusion of the first five-year follow-up, it showed similar pattern of results as the originals (Supplementary Table S2). (2) The results after exclusion of asthma cases with a higher possibility of COPD using the post-bronchodilator fixed ratio of FEV_1_/FVC or the lower limit of normal approach were similar to the original ones (Supplementary Tables S3 and S4). (3) Multiple imputation for missing data of all covariates including smoking showed comparable association estimates (Supplementary Table S5) compared with those before the imputation both in the primary cohort (Table 2) and in the cohort excluding the first 3-year follow-up (Table 3). (4) In the analysis using a negative control exposure (details are given in Supplementary Text, Figure and Table S6), migraine was inversely associated with heavy smoking in our study population. However, migraine was not associated with lung cancer incidence after adjustment for smoking, suggesting that our observed associations of active asthma and partially controlled asthma with lung cancer incidence were less likely biased by residual confounding due to smoking. (5) The results of competing risk analysis excluding the influence of death showed a similar trend as our main results with wider CIs due to many cases of death (n = 15,653) (Supplementary Table S7).

**Table 3:** The associations of asthma overall, asthma status and levels of asthma symptom control with lung cancer incidence after excluding the first three-year follow-up, the HUNT Study, 1995–97 to 2017 (n = 61,315).

					Crude^1^		Adjusted^2^	
Asthma overall			n/Cases	IR (per 1000 person-years)	HR	95% CI	HR	95% CI
No			58,211/834	0.88	1.00	Reference	1.00	Reference
Yes			3104/60	1.23	1.38	1.06–1.79	1.27	0.96–1.68
	Asthma status							
		Non-active asthma	1051/12	0.70	0.96	0.54–1.70	0.90	0.51–1.61
		Active asthma	2053/48	1.50	1.54	1.15–2.07	1.41	1.04–1.93
	Asthma symptom control^3^							
		Controlled	1152/15	0.78	0.96	0.57–1.60	0.98	0.58–1.66
		Partially controlled	1564/41	1.73	1.74	1.27–2.39	1.54	1.10–2.14

CI: Confidence interval; HR: Hazard ratio; IR: Incidence rate.

^1^Age was used as the time scale in the crude model.

^2^Adjusted for sex, body mass index, smoking [(never, former (< 10, 10–20, and > 20 pack-years (pyrs)), current (< 10, 10–20, and > 20 pyrs)], passive smoking, alcohol consumption, physical activity, total sitting time daily, education, economic difficulties, family history of cancer and allergic rhinitis. Age was used as the time scale. *Tvc* option of the *stcox* command in Stata was used to model the non-proportional hazards for sex, smoking and economic difficulties in the adjusted models.

^3^An “unknown” level of asthma symptom control with limited lung cancer cases (n = 4) is not shown.

## Discussion

In this prospective cohort study with 984 incident lung cancer cases during a median follow-up of 21.1 years, we did not observe a clear association between asthma overall and lung cancer incidence. However, partially controlled asthma was associated with an increased lung cancer incidence. Adults with active asthma showed a tendency of increased lung cancer incidence. There was no association of non-active asthma or controlled asthma with the lung cancer incidence.

Previous meta-analysis studies have suggested a positive association between asthma and lung cancer^2,4,5^. We did not observe a clear association between asthma overall and lung cancer incidence. One of the explanations for this discrepancy may be due to residual confounding by smoking. Many of the studies included in the meta-analyses did not thoroughly address the role of smoking in the association^6–8^. The best way to address confounding by smoking is to study the association among never smokers or with certain lung cancer histologic types. Rosenberger et al. found that the positive association between asthma and lung cancer became weaker in a sub-analysis among never smokers or when lung cancer was restricted to adenocarcinoma, a histologic type that is less strongly associated with smoking than other lung cancer subtypes^4^. We were not able to evaluate the associations in never smokers and with histologic types due to small number of lung cancer cases (e.g. there were only 56 lung cancer cases in the never smokers). Instead, we classified smoking status into detailed categories including pack-years in the adjusted model and performed sensitivity analyses such as multiple imputations and negative control exposure to address the possibility of residual confounding by smoking. Although we did not observe a clear association with asthma overall, we found that partially controlled asthma was associated with an increased incidence of lung cancer. Participants with active asthma had a tendency for increased lung cancer incidence. Analyses after multiple imputations for the missing data of all confounders including smoking showed similar pattern of results. The negative control exposure analysis using migraine also suggested that the observed associations were less likely biased by residual confounding from smoking, but residual confounding by smoking cannot completely be excluded. In line with our findings, Pirie and his colleagues included over half a million never smoking women from the UK Million Women Study who were followed up for more than 14 years and found that asthma requiring treatment was associated with an increased incidence of lung cancer^13^.

Active airway inflammation linked with active asthma or partially controlled asthma may be associated with the lung carcinogenic process through elevated levels of free radicals and reduced levels of antioxidants^3^, increased DNA damages and mutations^2^, and permanent abnormality of the airways^17^. In our study 29% of the active asthma patients were controlled. As there was no association between controlled asthma and incidence of lung cancer, this would dilute the association so that we only observed a tentative association between active asthma and incidence of lung cancer. Thus, levels of asthma symptom control may reflect the levels of inflammation better than the definition of active asthma. On the other hand, participants with partially controlled asthma were likely to visit their physicians. This might have led to an increased referral to x-ray of thorax and thus a greater chance for screening of lung cancer. In addition, 50% of the participants with partially controlled asthma in our study reported having used inhaled corticosteroid (ICS) regularly. Previous studies have reported an independently inverse association of the use of ICS with lung cancer risk^18,19^. Our study showed similar HRs for lung cancer incidence among adults with partially controlled asthma who used ICS compared with those who did not use ICS (data not presented). As we did not have information on the dosage or the patients’ compliance with ICS use, the potential influence of ICS on the risk of lung cancer warrants further investigation.

Our study is one of few prospective cohort studies that have investigated the potential associations of asthma overall, asthma status and symptom control with the incidence of lung cancer. We had a large and homogeneous study population and a long follow-up duration over 20 years. We also had information on a panel of potential confounders at baseline, which made it possible to minimize confounding. The information of lung cancer cases from the Cancer Registry of Norway is complete and accurate^20^. Misclassification of asthma due to early undiagnosed lung cancer, the so-called reverse causation, might not be an important problem in this study as we observed strengthened results after exclusion of the first three years of follow-up.

Our study has several limitations. First, misclassification of asthma was possible due to self-reporting. Nevertheless, self-reported asthma has been verified to be highly specific and reliable in many population studies^21^. In addition, the prevalence of asthma and active asthma (5.1% and 3.3% respectively) in our study was similar to a previous HUNT study using a slightly different definition of asthma^22^ and another Nordic study^23^. In our study 37% of the participants with asthma were symptom controlled, which was comparable with the findings from other European studies^14,15^. All information on asthma was collected at baseline long before the diagnosis of lung cancer. Thus, the misclassification of asthma was likely to be non-differential that in general would lead to an underestimated association. However, asthma symptoms fluctuate over time. The association estimates generated from the one-time measure of asthma symptoms from HUNT2 may not reflect the true effect of the varying asthma symptoms on lung cancer incidence. Second, COPD may be misdiagnosed as asthma due to similar symptoms, especially among elderly people. This can bias the association between asthma and lung cancer. However, we excluded participants with possible COPD according to the GOLD definition at baseline and further excluded asthma cases with higher possibility of COPD in the sensitivity analyses. Third, we did not have information on air pollution, radon, or occupational exposure to asbestos and other carcinogenic agents, which are also important risk factors for lung cancer^24^. However, except for two smallest municipalities having had mining industries previously, there was nearly no industrial pollution in the northern area of Trøndelag during the time of HUNT2 study^22^. The level of indoor radon in the county was shown to be in the safety range (< 200 Bq/m3) in the national measurement during 1999–2000^25^. No asbestos‐cement factories have existed in the county^26^. In any case, people with heavy exposure to asbestos should be minorities due to prohibition of importation and strict regulation to the use of asbestos in Norway since 1980^26^. At last, even if we have attempted to adjust for a large panel of potential confounders in our analyses, we cannot exclude the possibility of unknown confounding.

In conclusion, our study showed that participants with partially controlled asthma had an increased incidence of lung cancer. The finding suggested that proper control of asthma symptoms not only reduced asthma exacerbations but might also contribute to a reduced incidence of lung cancer.

## Methods

### Study design and population

The baseline data were derived from the second survey of The HUNT Study (HUNT2, 1995–1997). All adults aged 20 years or older living in the area of northern Trøndelag, Norway were invited to complete general questionnaires on health and lifestyle factors and undergo clinical examinations^27^.

A total of 65,227 adults (69% of the invited) participated in HUNT2. Every participant was followed up from the date of participation in HUNT2 until the date of first diagnosis of lung cancer, the date of death or emigration from Norway or the end of follow-up on December 31, 2017, whichever came first. Lung cancer diagnoses were obtained from the Cancer Registry of Norway. Information on vital status and emigration was obtained from the National Population Registry.

**Figure 1:**
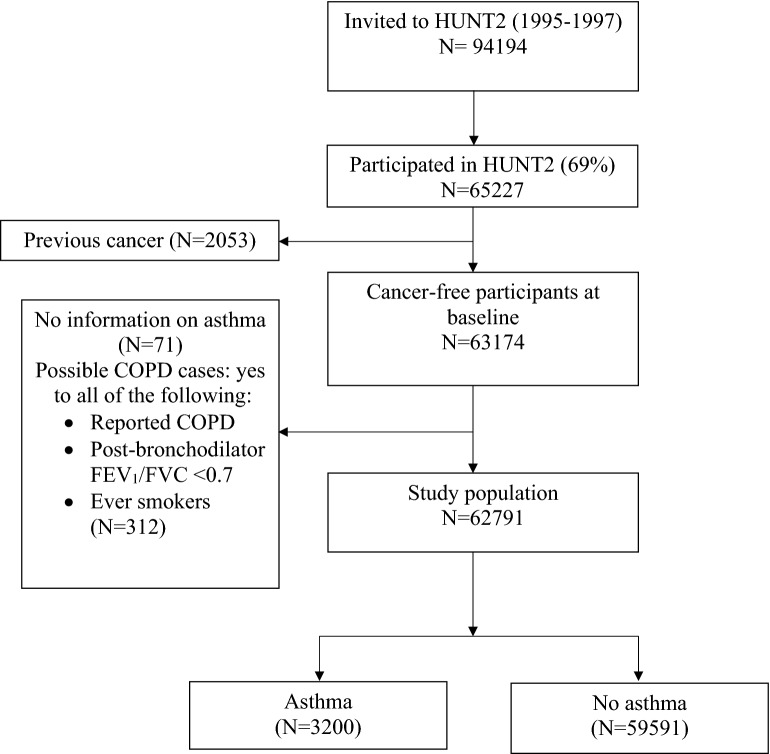
Flow chart of study participants.

We first excluded 2053 participants with previous cancer diagnoses before the baseline based on information from the Cancer Registry of Norway. Additionally, we excluded 71 participants without information on ever asthma. To minimize the influence by chronic obstructive pulmonary disease (COPD), we further excluded 312 adults who had possible COPD with all of the following criteria according to the Global Initiative for Chronic Obstructive Lung Disease (GOLD) definition^28^: reported doctor-diagnosed COPD, post-bronchodilator FEV_1_/FVC < 0.7 and ever smokers at baseline. Reported doctor-diagnosed COPD was defined based on the question “Have you been diagnosed as having chronic bronchitis or emphysema by a doctor?”. Lung function was measured by spirometry in HUNT2. The FEV_1_/FVC ratio was calculated from forced expiratory volume in 1 s (FEV_1_) and forced vital capacity (FVC)^28^. The post-bronchodilator fixed ratio (FEV_1_/FVC < 0.7) is the recommended spirometric criterion for diagnosing COPD^28^. This left 62,791 participants in the primary cohort for analyses (Fig. 1).

### Asthma definition

Detailed information on asthma history, symptoms and medication use was obtained from questionnaires^27^. Asthma was defined by affirmative answers to the following two questions: “Do you have, or have you had asthma?” in combination with “Have you been diagnosed as having asthma by a doctor?” (n = 3200). Asthma status was further categorized into active (n = 2110) and non-active (n = 1090) asthma. Participants were considered having active asthma if they confirmed symptoms of wheezing or reported using asthma medication in the last 12 months. The following four items were used to describe the level of asthma symptom control: 1) daytime symptoms more than twice weekly, 2) any night awakening, 3) need for reliever medications more than twice weekly or 4) any activity limitation based on the Global Initiative for Asthma (GINA) Global Strategy for Asthma Management and Prevention guidelines^29^. According to the GINA guidelines, controlled asthma refers to asthma without any of the above four items, partly controlled asthma refers to 1–2 items and uncontrolled asthma 3–4 items. We initially classified levels of asthma symptom control as controlled (n = 1170), partly controlled (n = 1227), uncontrolled (n = 395), and unknown (n = 408). Partly controlled and uncontrolled were collapsed into one category named “partially controlled” due to a small number of lung cancer cases in the uncontrolled category.

### Other baseline variables

Weight and height were measured by health professionals at clinical examination. Body mass index (BMI) was calculated as weight in kilograms divided by height squared in meter (kg/m^2^) and was grouped into three categories (< 25.0, 25.0–29.9, and ≥ 30.0 kg/m^2^) according to the recommendations of the World Health Organization (WHO)^30^. Based on information of smoking status and pack-years, participants were classified into the detailed categories of smoking: never, former (< 10, 10–20, and > 20 pack-years (pyrs)) and current (< 10, 10–20, and > 20 pyrs). Other covariates were categorized as: passive smoking (never, only childhood, only adulthood, and both), alcohol consumption (never, 1–4, and ≥ 5 times/month), physical activity (inactive, low, moderate, and high), total sitting time daily (0–4, 5–7, and ≥ 8 h), education (< 10, 10–12, and ≥ 13 years), economic difficulty (yes/no) and family history of cancer (yes/no). Participants were considered having allergic rhinitis if reporting having allergic rhinitis in combination with use of allergy medication or allergic symptoms to pollen or pets. Missing information on each of the aforementioned variables was included in the analyses as an “unknown” category.

### Ascertainment of lung cancer

By using the unique 11-digit personal identification number, participants’ information from HUNT2 was linked to the Cancer Registry of Norway^31^. The International Classification of Diseases version 10 (ICD-10) codes used for registration of lung cancer are C33-C34^31^. Data from the Cancer Registry of Norway are reasonably accurate and complete^32^.

### Statistical analysis

Baseline characteristics of the participants were presented by asthma categories (no asthma, non-active asthma, and active asthma). We used Cox proportional hazard models to examine the potential associations of asthma overall, asthma status, and levels of asthma symptom control with lung cancer incidence. Crude and adjusted hazard ratios (HRs) with 95% confidence intervals (CIs) were calculated. Age was used as the underlying time variable. Potential confounders were selected based on previous knowledge^33–36^ and directed acyclic graph (DAG). In the adjusted model, detailed categories of smoking status combined with pack-years was used to minimize confounding by smoking. The model also took account of sex, BMI, passive smoking, alcohol consumption, physical activity, total sitting time daily, education, economic difficulties, family history of cancer and allergic rhinitis. Allergic rhinitis was included in the model as a potential confounder because it commonly occurs together with asthma and has been reported to be inversely associated with lung cancer risk^34^.

We assessed the proportional hazards assumption by Schoenfeld Residuals for exposures and all covariates. Apart from sex, smoking and economic difficulties, other covariates did not show evidence against proportional hazards assumption. We therefore used the *tvc* option of the *stcox* command in Stata to model the non-proportional hazards for sex, smoking and economic difficulties.

We performed several sensitivity analyses to test the robustness of our findings: (1) To address reverse causality by existing but undiagnosed lung cancer, we excluded both the first three-year and five-year follow-up. (2) To further minimize the misclassification of COPD as asthma, we used two ways to exclude asthma cases with a higher possibility of COPD: we excluded asthma cases who had smoked ten pack-years or more and were older than 40 years when getting the asthma diagnosis and had (a) post-bronchodilator FEV_1_/FVC < 0.7 (fixed ratio criterion) (n = 104) or (b) post-bronchodilator FEV_1_/FVC z score < -1.64 (lower limit of normal criterion) (n = 75). The fixed ratio criterion is the mostly used approach to define airflow limitation, whereas the lower limit of normal criterion overcomes the overestimation of the number of COPD among elderly^28^. (3) To address residual confounding by smoking and other covariates due to information missing, we conducted multivariable chained imputation with fully conditional specification (m = 10 imputed datasets) for the missing data of all covariates based on the assumption of missing at random. (4) To further address residual confounding by smoking, we performed analysis using migraine as a negative control exposure. This negative control exposure should be associated with the same confounder as the main exposure but not causally associated with the outcome ^37^. The aim of analysis using a negative control exposure is to identify residual confounding that may have resulted in invalid causal inference for the main exposure-outcome association^37^. Previous study suggested that migraine was associated with smoking^38^ but not with lung cancer. If we observed no association between migraine and lung cancer after adjustment for smoking, it indicated that the main exposure-outcome (asthma-lung cancer) association was less likely resulted from residual confounding by smoking. (5) To deal with possible competing risk due to death, a competing risk analysis based on Fine-Gray model was used^39^. All statistical analyses were performed with STATA/SE 15.1 (College Station, TX, USA).

## Ethics approval

The study was approved by the Regional Committee for Medical and Health Research Ethics of South-East Norway (2015/78/REC South-East). All participants signed informed written consent on participation in HUNT. The study was performed in accordance with the ethical standards as laid down in the 1964 Declaration of Helsinki and its later amendments or comparable ethical standards.

## Supplementary Information

Supplementary Information

## Data Availability

Data from the HUNT Study is available on request to the HUNT Data Access Committee (hunt@ medisin.ntnu.no) when is used in research projects. The HUNT data access information describes the policy regarding data availability (https://www.ntnu.edu/hunt/data).
