# Supplementary material for: Asthma and asthma symptom control in relation to incidence of lung cancer in the HUNT study
Source: Sci Rep. 2021 Feb 25;11:4539. doi: 10.1038/s41598-021-84012-3 (PMC7907333; doi:10.1038/s41598-021-84012-3)
Supplement: Supplementary file 1 — Supplementary Information [file 41598_2021_84012_MOESM1_ESM.pdf]

Asthma and asthma symptom control in relation to incidence of lung cancer in the HUNT Study

Lin Jiang [1,] *

Yi-Qian Sun [2,3,4]

Arnulf Langhammer [5,6]

Ben Michael Brumpton [7,8,9]

Yue Chen [10]

Tom IL Nilsen [1,11]

Linda Leivseth [12]

Sissel Gyrid Freim Wahl [2,3]

Xiao-Mei Mai [1]

[1] Department of Public Health and Nursing, Faculty of Medicine and Health Science, Norwegian University of Science and Technology, Trondheim, Norway.

[2] Department of Clinical and Molecular Medicine, Faculty of Medicine and Health Science, Norwegian University of Science and Technology, Trondheim, Norway.

[3] Department of Pathology, Clinic of Laboratory Medicine, St. Olavs Hospital, Trondheim University Hospital, Trondheim, Norway.

[4] TkMidt-Center for Oral Health Services and Research, Mid-Norway, Trondheim, Norway

[5] HUNT Research Centre, Department of Public Health and Nursing, Norwegian University of Science and Technology, Levanger, Norway

[6] Levanger Hospital, Nord-Trøndelag Hospital Trust, Levanger, Norway

[7] Clinic of Thoracic and Occupational Medicine, St. Olavs Hospital, Trondheim University Hospital, Trondheim, Norway

[8] K.G. Jebsen Centre for Genetic Epidemiology, Department of Public Health and Nursing, Norwegian University of Science and Technology, Trondheim, Norway.

[9] MRC Integrative Epidemiology Unit, University of Bristol, Bristol, United Kingdom.

[10] School of Epidemiology and Public Health, Faculty of Medicine, University of Ottawa, Ottawa, Canada.

[11] Clinic of Anesthesia and Intensive Care, St. Olavs Hospital, Trondheim University Hospital, Trondheim, Norway

[12] Centre for Clinical Documentation and Evaluation (SKDE), Northern Norway Regional Health Authority, Tromsø, Norway

*Corresponding author

Address for correspondence: Department of Public Health and Nursing, Faculty of Medicine and Health Science, Norwegian University of Science and Technology (NTNU), Postbox 8905, MTFS, N-7491 Trondheim, Norway.

E-mail: lin.jiang@ntnu.no

Supplementary Table S1. Distribution of baseline characteristics according to levels of asthma symptom control in the HUNT2 Study, 1995-1997 (n=62791).

Variables	No asthma	Controlled asthma	Partially controlled asthma	Unknown
**Number of subjects**	**59591**	**1170**	**1622**	**408**
Age (years)	49.5±17.0	47.0±16.2	52.1±17.5	48.5±18.8
Body mass index (kg/m^2)	26.3±4.0	26.7±4.3	27.4±5.0	27.3±5.2
**Number of lung cancer cases (%)**	**921(1.6)**	**15(1.3)**	**42 (2.6)**	**6 (1.5)**
Sex, % (women/men)	53.0/47.0	53.4/46.6	55.0/45.0	55.6/44.4
Allergic rhinitis, % (no/yes/unknown)	70.5/5.5/24.0	41.1/45.2/13.7	34.5/51.2/14.4	36.5/47.8/15.7
Smoking status, % (never/current/former/unknown)	43.0/28.6/26.3/2.2	41.1/27.4/29.8/1.7	36.3/28.2/33.2/2.3	39.2/32.3/25.5/2.9
Passive smoking, % (never/ever/unknown)	18.5/79.6/1.9	16.9/81.8/1.3	14.4/83.5/2.0	12.8/85.5/1.7
Alcohol consumption (times/month), % (never/≥1/unknown)	34.6/56.9/8.6	31.9/60.5/7.6	40.5/51.1/8.5	39.7/51.7/8.6
Physical activity, % (inactive[1]/active[2]/unknown)	21.6/48.0/30.4	21.9/50.5/27.6	23.8/44.3/31.9	26.5/50.7/22.8
Total sitting time daily (hours), % (<8/≥8/unknown)	48.1/27.8/24.1	54.7/33.0/12.3	53.8/32.7/13.5	50.7/34.3/15.0
Education (years), % (<10/≥10/unknown)	34.0/61.0/5.1	30.7/65.4/3.9	40.2/53.1/6.7	36.5/56.9/6.6
Economic difficulties, % (no/yes/unknown)	48.0/21.1/30.9	56.8/26.1/17.2	45.3/31.8/23.0	40.0/34.8/25.3
Family history of cancer, % (no/yes)	75.0/25.0	70.8/29.2	68.6/31.4	76.5/23.5

HUNT: Nord-Trøndelag Health Study.

Data are given as mean ± standard deviation or percentage of subjects in each asthma category.

[1] Inactive: no physical activity or only light physical activity ≤2 h per week.

[2] Active: physical activity level from low to high.

Supplementary Table S2. The associations of asthma overall, asthma status and levels of asthma symptom control with lung cancer incidence after excluding the first five-year follow-up, the HUNT Study, 1995-97 to 2017 (n=59944)

					Crude[1]		Adjusted[2]	
Asthma overall			n/Cases	IR (per 1000 person-years)	HR	95% CI	HR	95% CI
No			56941/784	0.94	1.00	Reference	1.00	Reference
Yes			3003/54	1.26	1.33	1.01-1.75	1.22	0.91-1.64
	Asthma status							
		Non-active asthma	1020/12	0.80	1.02	0.58-1.81	0.95	0.53-1.70
		Active asthma	1983/42	1.51	1.46	1.07-1.99	1.33	0.96-1.85
	Asthma symptom control[3]							
		Controlled	1133/15	0.89	1.01	0.61-1.68	1.02	0.60-1.72
		Partially controlled	1495/35	1.69	1.61	1.15-2.26	1.43	1.00-2.04

CI: Confidence interval; HR: Hazard ratio; IR: Incidence rate.

[1] Age was used as the time scale in the crude model.

[2] Adjusted for sex, body mass index, smoking [(never, former (<10, 10-20, and >20 pack-years (pyrs)), current (<10, 10-20, and >20 pyrs)], passive smoking, alcohol consumption, physical activity, total sitting time daily, education, economic difficulties, family history of cancer and allergic rhinitis. Age was used as the time scale. *Tvc* option of the *stcox* command in Stata was used to model the non-proportional hazards for sex, smoking and economic difficulties in the adjusted models.

[3] An “unknown” level of asthma symptom control with limited lung cancer cases (n=4) is not shown.

Supplementary Table S3. The associations of asthma overall, asthma status and levels of asthma symptom control with lung cancer incidence after excluding participants with asthma who also had post bronchodilator FEV_1/FVC <0.7, smoking package years ≥10 and age at asthma diagnosis >40 years, the HUNT Study, 1995-97 to 2017 (n=62687)

					Crude[1]		Adjusted[2]	
Asthma overall			n/Cases	IR (per 1000 person-years)	HR	95% CI	HR	95% CI
No			59591/921	0.82	1.00	Reference	1.00	Reference
Yes			3096/55	0.96	1.19	0.90-1.56	1.20	0.90-1.60
	Asthma status							
		Non-active asthma	1085/14	0.69	1.01	0.60-1.72	0.97	0.57-1.66
		Active asthma	2011/41	1.11	1.26	0.92-1.72	1.31	0.94-1.82
	Asthma symptom control[2]							
		Controlled	1148/13	0.58	0.78	0.45-1.35	0.86	0.49-1.50
		Partially controlled	1540/36	1.31	1.46	1.04-2.03	1.46	1.03-2.07

CI: confidence interval; HR: hazard ratio; IR: incidence rate.

[1] Age was used as the time scale in the crude model.

[2] Adjusted for sex, body mass index, smoking [(never, former (<10, 10-20, and >20 pack-years (pyrs)), current (<10, 10-20, and >20 pyrs)], passive smoking, alcohol consumption, physical activity, total sitting time daily, education, economic difficulties, family history of cancer and allergic rhinitis. Age was used as the time scale. *Tvc* option of the *stcox* command in Stata was used to model the non-proportional hazards for sex, smoking and economic difficulties in the adjusted model.

[3] An “unknown” level of asthma symptom control is not shown due to limited lung cancer cases (n=6).

Supplementary Table S4. The associations of asthma overall, asthma status and levels of asthma symptom control with lung cancer incidence after excluding participants with asthma who also had post bronchodilator FEV_1/FVC z score <-1.64, smoking package years ≥10 and age at asthma diagnosis >40 years, the HUNT Study, 1995-97 to 2017 (n=62716)

					Crude[1]		Adjusted[2]	
Asthma overall			n/Cases	IR (per 1000 person-years)	HR	95% CI	HR	95% CI
No			59591/921	0.82	1.00	Reference	1.00	Reference
Yes			3125/58	1.01	1.23	0.94-1.61	1.21	0.91-1.60
	Asthma status							
		Non-active asthma	1087/14	0.69	1.01	0.60-1.71	0.96	0.56-1.64
		Active asthma	2038/44	1.18	1.32	0.98-1.79	1.32	0.96-1.82
	Asthma symptom control[2]							
		Controlled	1157/13	0.58	0.77	0.45-1.34	0.82	0.47-1.44
		Partially controlled	1560/39	1.40	1.55	1.12-2.13	1.49	1.06-2.08

CI: confidence interval; HR: hazard ratio; IR: incidence rate.

[1] Age was used as the time scale in the crude model.

[2] Adjusted for sex, body mass index, smoking [(never, former (<10, 10-20, and >20 pack-years (pyrs)), current (<10, 10-20, and >20 pyrs)], passive smoking, alcohol consumption, physical activity, total sitting time daily, education, economic difficulties, family history of cancer and allergic rhinitis. Age was used as the time scale. *Tvc* option of the *stcox* command in Stata was used to model the non-proportional hazards for sex, smoking and economic difficulties in the adjusted model.

[3] An “unknown” level of asthma symptom control is not shown due to limited lung cancer cases (n=6).

Supplementary Table S5. The associations of asthma overall, asthma status and levels of asthma symptom control with lung cancer incidence after multiple imputations, the HUNT Study, 1995-97 to 2017 in the primary analysis cohort and in cohort after excluding the first three-year follow-up

			Primary cohort (n=62791)		Cohort after excluding the first 3-year follow-up (n=61315)	
Asthma overall			Adjusted[1] HR	95% CI	Adjusted[1] HR	95% CI
No			1.00	Reference	1.00	Reference
Yes			1.14	0.85-1.52	1.21	0.91-1.62
	Asthma status					
		Non-active asthma	0.90	0.52-1.56	0.86	0.48-1.55
		Active asthma	1.23	0.90-1.70	1.35	0.98-1.86
	Asthma symptom control[2]					
		Controlled	0.86	0.51-1.47	0.95	0.56-1.60
		Partially controlled	1.33	0.95-1.87	1.46	1.04-2.06

CI: confidence interval; HR: hazard ratio; IR: incidence rate.

[1] Adjusted for sex, body mass index, smoking [(never, former (<10, 10-20, and >20 pack-years (pyrs)), current (<10, 10-20, and >20 pyrs)], passive smoking, alcohol consumption, physical activity, total sitting time daily, education, economic difficulties, family history of cancer and allergic rhinitis. Age was used as the time scale. *Tvc* option of the *stcox* command in Stata was used to model the non-proportional hazards for sex, smoking and economic difficulties in the adjusted models.

[2] An “unknown” level of asthma symptom control is not shown due to limited lung cancer cases (n=6).

Supplementary Text. Analysis using negative control exposure

The aim of analysis using a negative control exposure is to identify residual confounding that may have resulted in invalid causal inference for the main exposure-outcome association[1]. In the current study, we used “migraine” as the negative control exposure to detect residual confounding by smoking in the observed asthma-lung cancer association (Supplementary Figure). “Migraine” was chosen as the negative control exposure was because it is associated with the confounder (smoking)[2,3], but not causally associated with the outcome (lung cancer). We expected to observe a null association between migraine and lung cancer after adjustment for smoking, suggesting that the observed asthma-lung cancer association was less likely biased by the residual confounding of smoking.

Based on our study population (n=62791), we excluded participants without information on headache. This left 49945 participants to study the relationship between migraine and lung cancer incidence. Participants with migraine were those who answered yes to the question “Have you suffered from headache during the last 12 months?” and specified the type of headache as “migraine”, and the rest were regarded as no migraine. We adjusted for smoking status in model 1. In model 2, we adjusted for the same confounders as in our primary study (Supplementary Table S6).

The negative control exposure analysis showed that fewer participants with migraine were heavy smokers (> 20 pack-years) than participants without migraine (6.1% vs 10.3%). There was an inverse association between migraine and lung cancer incidence without adjustment for smoking (crude HR 0.61, 95% CI 0.43-0.86). After adjustment for smoking the association between migraine and lung cancer became less clear (HR 0.75, 95% CI 0.53-1.06), and additional adjustment for the same confounders as in the primary study did not have material changes in the result. This indicated that our observed associations of active asthma and partially controlled asthma with increased lung cancer incidence were less likely biased by residual confounding due to smoking.

Supplementary Figure. DAG for asthma (as the main exposure), migraine (as the negative control exposure) and incidence of lung cancer (as the outcome)

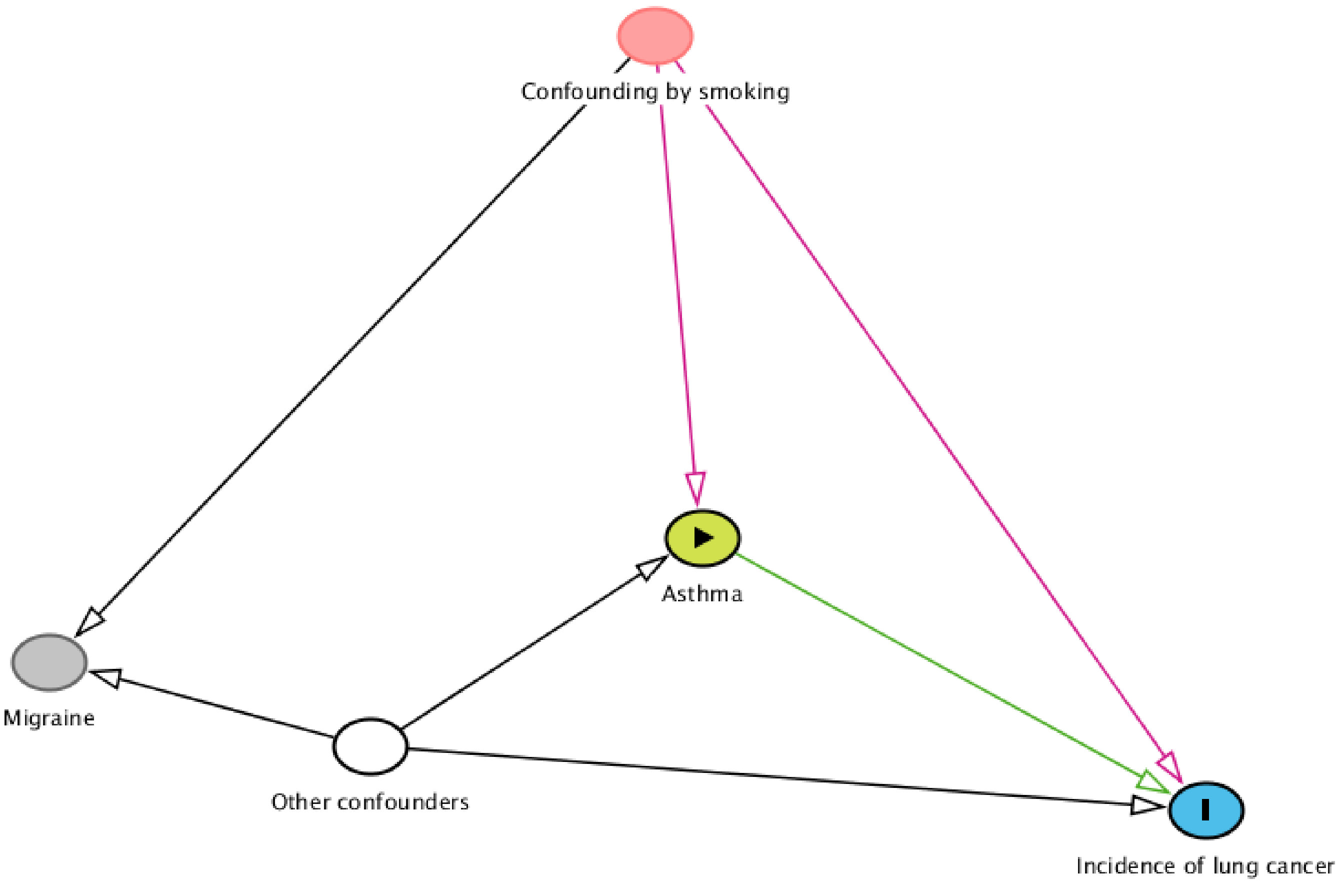

Figure legend. This DAG was created by using DAGitty V 3.0[4] (http://www.dagitty.net/dags.html).

Supplementary Table S6. Negative control using migraine as an alternative exposure to address residual confounding by smoking for the association of asthma with lung cancer incidence, the HUNT Study, 1995-97 to 2017 (N=49945)

				Crude[1]		Adjusted[2]		Adjusted[3]	
	n	Cases	IR (per 1000 person-years)	HR	95% CI	HR	95% CI	HR	95% CI
Non-migraine	45750	721	0.83	1.00	Reference	1.00	Reference	1.00	Reference
Migraine	4195	35	0.41	0.61	0.43-0.86	0.75	0.53-1.06	0.75	0.53-1.05

CI: confidence interval; HR: hazard ratio; IR: incidence rate.

[1] Age was used as the time scale in the crude model.

[2] Adjusted for smoking [(never, former (<10, 10-20, and >20 pack-years (pyrs)), current (<10, 10-20, and >20 pyrs)]. Age was used as the time scale.

[3] The same confounders as for the association between asthma and lung cancer incidence: sex, body mass index, smoking [(never, former (<10, 10-20, and >20 pack-years (pyrs)), current (<10, 10-20, and >20 pyrs)], passive smoking, alcohol consumption, physical activity, total sitting time daily, education, economic difficulties, family history of cancer and allergic rhinitis. Age was used as the time scale.

Supplementary Table S7. The associations of asthma overall, asthma status and levels of asthma symptom control with lung cancer incidence taking into account competing risk due to death, the HUNT Study, 1995-97 to 2017 (n=62791)

Asthma overall			n/Cases	IR (per 1000 person-years)	Adjusted[1] SHR	95% CI
No			59591/921	0.82	1.00	Reference
Yes			3200/63	1.08	1.10	0.84-1.44
	Asthma status					
		Non-active asthma	1090/14	0.69	0.86	0.50-1.47
		Active asthma	2110/49	1.28	1.20	0.88-1.62
	Asthma symptom control[2]					
		Controlled	1170/15	0.66	0.86	0.51-1.46
		Partially controlled	1622/42	1.47	1.26	0.92-1.74

CI: confidence interval; IR: incidence rate; SHR: sub-distribution hazard ratio.

[1] Adjusted for sex, body mass index, smoking [(never, former (<10, 10-20, and >20 pack-years (pyrs)), current (<10, 10-20, and >20 pyrs)], passive smoking, alcohol consumption, physical activity, total sitting time daily, education, economic difficulties, family history of cancer and allergic rhinitis. Age was used as the time scale. *Tvc* option of the *stcox* command in Stata was used to model the non-proportional hazards for sex, smoking and economic difficulties in the adjusted model.

[2] An “unknown” level of asthma symptom control is not shown due to limited lung cancer cases (n=6).

References

1.Lipsitch, M., Tchetgen, E.T. & Cohen, T. Negative controls: a tool for detecting confounding and bias in observational studies. *Epidemiology (Cambridge, Mass.)* **21**, 383 (2010).
2.Taylor, F.R. Tobacco, Nicotine, and Headache. *Headache* **55**, 1028-1044 (2015).
3.Aamodt, A., Stovner, L., Hagen, K., Bråthen, G. & Zwart, J. Headache prevalence related to smoking and alcohol use. The Head-HUNT Study. *Eur. J. Neurol.* **13**, 1233-1238 (2006).
4.Textor, J., van der Zander, B., Gilthorpe, M.S., Liśkiewicz, M. & Ellison, G.T. Robust causal inference using directed acyclic graphs: the R package ‘dagitty’. *Int. J. Epidemiol.* **45**, 1887-1894 (2016).
